# Phenytoin Decreases Pain-like Behaviors and Improves Opioid Analgesia in a Rat Model of Neuropathic Pain

**DOI:** 10.3390/brainsci13060858

**Published:** 2023-05-25

**Authors:** Magdalena Kocot-Kępska, Katarzyna Pawlik, Katarzyna Ciapała, Wioletta Makuch, Renata Zajączkowska, Jan Dobrogowski, Anna Przeklasa-Muszyńska, Joanna Mika

**Affiliations:** 1 Department of Pain Research and Treatment, Jagiellonian University Medical College, 31-501 Krakow, Poland; jan.dobrogowski@uj.edu.pl (J.D.); a.przeklasa-muszynska@uj.edu.pl (A.P.-M.); 2 Department of Pain Pharmacology, Maj Institute of Pharmacology Polish Academy of Sciences, 31-343 Krakow, Poland; pawlik@if-pan.krakow.pl (K.P.); kat.ciapala@gmail.com (K.C.); makuch@if-pan.krakow.pl (W.M.); 3 Department of Interdisciplinary Intensive Care, Jagiellonian University Medical College, 30-688 Krakow, Poland; renata.zajaczkowska@uj.edu.pl

**Keywords:** phenytoin, neuropathic pain, morphine, glia, neuroinflammation

## Abstract

Neuropathic pain remains a clinical challenge due to its complex and not yet fully understood pathomechanism, which result in limited analgesic effectiveness of the management offered, particularly for patients with acute, refractory neuropathic pain states. In addition to the introduction of several modern therapeutic approaches, such as neuromodulation or novel anti-neuropathic drugs, significant efforts have been made in the repurposing of well-known substances such as phenytoin. Although its main mechanism of action occurs at sodium channels in excitable and non-excitable cells and is well documented, how the drug affects the disturbed neuropathic interactions at the spinal cord level and how it influences morphine-induced analgesia have not been clarified, both being crucial from a clinical perspective. We demonstrated that single and repeated systemic administrations of phenytoin decreased tactile and thermal hypersensitivity in an animal model of neuropathic pain. Importantly, we observed an increase in the antinociceptive effect on thermal stimuli with repeated administrations of phenytoin. This is the first study to report that phenytoin improves morphine-induced antinociceptive effects and influences microglia/macrophage activity at the spinal cord and dorsal root ganglion levels in a neuropathic pain model. Our findings support the hypothesis that phenytoin may represent an effective strategy for neuropathic pain management in clinical practice, particularly when combination with opioids is needed.

## 1. Introduction

Neuropathic pain (NP) is defined by the International Association for the Study of Pain (IASP) as pain caused by a lesion or disease of the somatosensory nervous system [1]. NP is not a particular disease itself but a clinical condition that is caused by a variety of different diseases and lesions affecting the nervous system at the peripheral and central levels. Nerve damage induces an extensive response in either the neuronal or immune system, resulting in close interplay among neuronal, immune and glial cells, which has been observed in both animal and human research [2]. In clinical practice, NP may thus be considered a complex neuroimmune disorder with multiple neuronal and non-neuronal mechanisms involved, either in the periphery or centrally [2,3]. The complex and not yet fully understood pathogenesis of NP contributes to the limited effectiveness of recommended treatment in clinical practice, as only half of patients can achieve 30–50% pain relief, while the remainder unfortunately cannot be helped [3]. To improve the quality of pain control in NP patients, symptomatic treatment, including pharmacotherapy, should be aimed at neural and non-neural processes simultaneously, preferably with drugs with pleiotropic properties or with combination therapy [4,5].

The substance that we believe may meet the pleiotropic drug criteria is phenytoin. Phenytoin (PHT), a hydantoin derivate, is a first-generation anticonvulsant drug that has been used clinically for over 80 years in the treatment of partial seizures or tonic–clonic (grand mal) seizures, as well as in acute treatment of generalized status epilepticus. After implementation in clinical practice, PHT has been effectively used as a co-analgesic in the treatment of several neuropathic pain syndromes, including trigeminal neuralgia [6], glossopharyngeal neuralgia [7], cancer-related pain [8,9], diabetic neuropathy [10], central pain [11,12], complex regional pain syndrome [13], Fabry disease [14] and peripheral neuropathic pain [15]. Currently, in pain medicine, systemic PHT is recommended and utilized in patients with trigeminal neuralgia as monotherapy or add-on therapy, particularly in patients with trigeminal neuralgia crisis [16,17] and in patients with other severe NP syndromes when immediate and effective control of pain is necessary [15,18]. Although its usage in clinical practice is diminished due to adverse effects, PHT is enumerated on the World Health Organizations List of Essential Medicines; it is cost-effective and easily available in several formulations (per os, parenteral) for out- and inpatient settings, which makes the drug a viable clinical option as monotherapy or in combination with analgesics, e.g., opioids, particularly in patients with acute, severe and refractory NP [19].

PHT acts via multiple mechanisms, being of key importance either in epilepsy or in neuropathic pain pathogenesis. PHT non-selectively blocks voltage-dependent sodium channels (Nav) 1.1–1.7 in a voltage- and frequency-dependent manner, escalating the threshold and reducing the duration of firing in excitable cells, with weak suppression of sodium currents during the periods between firings [20,21]. Studies in vivo and in vitro have also demonstrated that PHT blocks Nav isoforms expressed in non-excitable cells: Nav 1.5 in cancer cells, resulting in reduced tumor growth and metastasis [22,23]; and Nav 1.1, 1.5 and 1.6 expressed in microglia, resulting in reduced phagocytosis, migration and secretion of the cytokines IL-1α, IL-1β and TNF-α [24]. Since isoforms of Nav are widely expressed in other non-excitable cells, such as astroglia [25], Schwann cells [26], macrophages [27], lymphocytes [28], dendritic cells [29], fibroblasts [30], osteoblasts [31], keratinocytes [32] and epithelial cells [33], one cannot exclude potential interactions of PHT with Nav expressed by these cells and their contribution to the antinociceptive mechanisms of PHT—in particular, by influencing non-neuronal cells. Preclinical studies have revealed several additional mechanisms of action of PHT at the molecular level, potentially contributing to its antinociceptive and anti-inflammatory properties, such as interaction with voltage gated calcium channels (VGCC) type L [34] and type T [35] and GABAergic signaling [36] and the reduction of matrix metalloproteinase activity [37].

In vivo and in vitro studies have demonstrated that PHT may influence the inflammatory process by several mechanisms, e.g., suppressing lymphocyte activity [38,39,40], decreasing inflammatory cell infiltration [41,42,43,44], reducing microglial activation [41] and influencing the release of pro- and anti-inflammatory cytokines [45,46,47,48,49]. However, it is still unclear how PHT affects neuroimmune interactions at the spinal cord level in neuropathic pain models, as the aforementioned findings come mainly from studies in models of inflammation per se, epilepsy and wound healing. To date, it is well known that peripheral nerve injuries induce a pronounced immune reaction within the spinal cord, largely governed by microglia/macrophage activation in the spinal cord and dorsal root ganglia (DRGs) [2,50,51].

Therefore, one of the aims of this study was to assess whether and how PHT affects spinal microglia/macrophages in a model of neuropathic pain and its influence on pain-related behavior. To the best of our knowledge, experimental studies have not yet evaluated the efficacy of the combined use of PHT with opioids in NP models; therefore, our study aimed to verify the impact of PHT on opioid-induced analgesia. We first examined the influence of a single intraperitoneal (*i.p.*) administration of PHT on already fully developed tactile and thermal hypersensitivity in rats following chronic constriction injury (CCI) of the sciatic nerve (Bennett model). Second, we studied how preemptive and repeated (for 7 days) *i.p.* PHT injections influenced the development of pain-related behavior following CCI and its concurrent impact on the protein level of IBA-1, the cellular marker of microglia/macrophages in the spinal cord and macrophages in the DRG, as determined by Western blotting. Our third goal was to assess how *i.p.* PHT administration influenced morphine analgesia in the neuropathic pain model. The results obtained in our experiment may translate into the clinic, as we may gain evidence on the complex mechanism of action of PHT and its potentiation of opioid-induced analgesia, which can justify the broader clinical utilization of PHT as monotherapy or in combination with opioid analgesics in patients with refractory, severe neuropathic pain syndromes, where neurogenic inflammation predominates.

## 2. Materials and Methods

### 2.1. Animals

Male Wistar rats (275–300 g) provided by Charles River Laboratories International, Inc. (Sulzfeld, Germany) were used for this study. Animals were housed in cages covered with sawdust with food and water available ad libitum under a standard 12-h/12-h light/dark cycle (lights on at 06.00 a.m.). The animals were allowed to acclimate to the environment for approximately 5 min before the behavioral tests. Experiments were carried out according to the recommendations and standards of IASP and the National Institutes of Health (NIH) Guide for the Care and Use of Laboratory Animals and were approved by the Ethical Committee of the Maj Institute of Pharmacology of the Polish Academy of Sciences (Krakow, Poland, LKE: 96/2022). According to the 3R policy, the number of rats was reduced to the necessary minimum. Animal studies are reported in compliance with the ARRIVE guidelines.

### 2.2. Sciatic Nerve Surgery

Chronic constriction injury (CCI) of the sciatic nerve in rats was performed under sodium pentobarbital anesthesia (60 mg/kg, *i.p.*) using the procedure described by Bennett and Xie [52] and our previously published papers [53,54,55]. An incision was made below the hip bone, and the *biceps femoris* and *gluteus superficialis* were separated. The exposed right sciatic nerve was loosely tied four times with 1-mm spacing using 4/0 silk ligatures until a brief twitch in the operated hind limb appeared. After the procedure, the rats developed long-lasting thermal and tactile hypersensitivity.

### 2.3. Drug Administration

The following drugs were used in the present study: phenytoin (PHT; Pfizer, Holland; solution for injection) and morphine hydrochloride (M; Fagron, Krakow, Poland). Before intraperitoneal administration (*i.p.*), all the drugs were dissolved in physiological saline solution (0.9% NaCl). The control group received a vehicle (V, 0.9% NaCl) according to the same protocols. No adverse side effects of PHT or M treatment were observed during the experiments. Behavioral experiments were performed between 8 a.m. and 12 p.m. PHT was administered in three experimental schedules. In the first set of experiments, *i.p.* PHT was given at 3 doses: 10, 30, 60 mg/kg in a single dose on Day 7 after CCI. One hour after the vehicle or PHT injection, von Frey and cold plate tests were performed. This time point was chosen based on the literature showing that after-1-h PHT shows the best analgesia compared to other models of pain, the partial nerve ligation, visceral and formalin [56]. In the second set of trials, *i.p.* PHT was given at a dose of 30 mg/kg 16 and 1 h before CCI surgery and then once daily for the following 7 days after CCI—this scheme of administration was used already in our previous studies. Moreover, this regimen was chosen because many studies indicate that it is easier to silence glia before it is activated [53,57,58,59,60]. Behavioral tests were performed 1 h after vehicle or PHT injection on Days 2 and 7 after CCI. In the third set of experiments, a single *i.p.* dose of PHT 30 mg/kg was administered on Day 8 after CCI, and then 0.5 h after PHT delivery, morphine (10 mg/kg, *i.p.*) was injected. The von Frey and cold plate tests were conducted 0.5 h after opioid injection (1 h after PHT administration).

### 2.4. Behavioral Tests

#### 2.4.1. Tactile Hypersensitivity (Von Frey Test)

The von Frey apparatus (Dynamic Plantar Anesthesiometer, Cat. No. 37400, Ugo Basile, Gemonio, Italy) was used to measure mechanical hypersensitivity in rats, as previously described. The CCI-exposed animals were placed in plastic cages with a wire mesh floor 5 min before the experiment [61,62]. The rats were able to move freely on the surface. The machine’s touch stimulator was moved under the operated right hind limb, and the reaction of the animal to the stimulus was measured automatically. The strength of the von Frey touch stimulator was up to 26 g. In naive animals, the reaction of the right paw was measured.

#### 2.4.2. Thermal Hypersensitivity (Cold Plate Test)

Thermal hypersensitivity was measured using a cold plate apparatus (Cold/Hot Plate Analgesia Meter No. 05044 Columbus Instruments, Columbus, OH, USA), as previously described. Each animal was placed on a cold surface (5°) and kept there until it lifted its hind paw. The cutoff latency was 30 s, as described previously [61,62]. The rats were placed on the cold plate, and the time until the injured paw was lifted was recorded. In CCI-exposed rats, the injured (right) paw always reacted first; therefore, the effect of the treatment is presented as the reaction of the ipsilateral hind paw. In naive animals, reactions of both paws were observed.

### 2.5. Western Blotting

Tissues from ipsilateral spinal cord segments (L4-L6) and DRG were collected from naive and CCI-exposed rats after decapitation on Day 7 after CCI, 6 h after the last drug administration. Then, samples were placed into RIPA buffer supplemented with a protease inhibitor cocktail (Sigma-Aldrich, St. Louis, MO, USA), homogenized and cleared via centrifugation (14,000 rpm, 30 min, 4 °C). The total protein concentration was measured using the bicinchoninic acid method. The obtained samples (10 µg of protein) were heated in a mix of loading buffer (4× Laemmli Buffer, Bio-Rad, Warsaw, Poland) with 2-mercaptoethanol (Bio-Rad) for 8 min at 98 °C. Electrophoresis was performed using 4–15% Criterion™ TGX™ precast polyacrylamide gels (Bio-Rad). Next, the proteins were transferred (semidry transfer, 25 V, 30 min) to polyvinylidene fluoride membranes (Bio-Rad) and then blocked for 1 h at room temperature (RT) using 5% bovine serum albumin (Sigma-Aldrich St. Louis, MI, USA) in Tris-buffered saline containing 0.1% Tween-20 (TBST). Later, the membranes were washed with TBST and incubated overnight (4 °C) with the following primary antibodies: rabbit anti-IBA-1 (1:500; Novus) and mouse anti-GAPDH (1:5000, Merck, Darmstadt, Germany). The next day, the membranes were washed with TBST and incubated for 1 h at RT with horseradish peroxidase-conjugated anti-rabbit or anti-mouse secondary antibodies (1:5000, Vector Laboratories, Burlingame, CA, USA) diluted in buffer from a SignalBoost™ Immunoreaction Enhancer Kit (Merck). Proteins were detected using Clarity™ Western ECL Substrate (Bio-Rad) and visualized using a Fujifilm LAS-4000 FluorImager system. Fujifilm MULTI GAUGE software (Tokyo, Japan) was used to estimate the intensities of immunoreactive bands.

### 2.6. Statistical Analysis

Data from behavioral studies are presented as the mean grams or seconds ± SEM. In the case of biochemical analyses, the results of Western blot are presented as the fold change relative to the control (naive) ± SEM. The intergroup differences were analyzed using one-way analysis of variance (ANOVA) followed by Bonferroni’s post hoc test for multiple comparisons. All data were analyzed using GraphPad Prism 9.0 software (GraphPad, San Diego, CA, USA).

## 3. Results

### 3.1. The Influence of a Single i.p. Phenytoin Administration on Pain-Related Behavior Measured on Day 7 after Chronic Constriction Injury of the Sciatic Nerve in Rats

**Figure 1:**
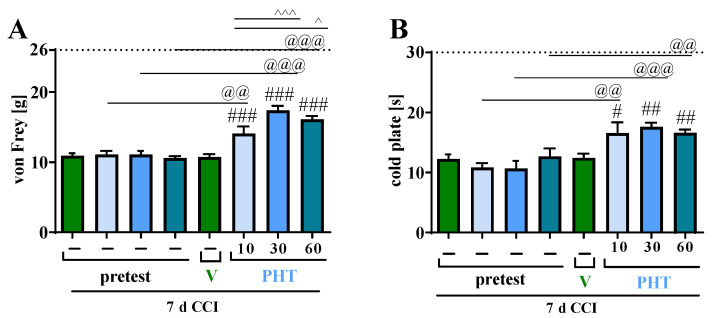
Effect of a single administration of PHT (10, 30, 60 mg/kg, intraperitoneally) on pain-related behaviors in rats (von Frey test—(**A**) and cold plate test—(**B**)) 1 h after drug injection on Day 7 after chronic constriction injury of the sciatic nerve. The horizontal dotted line represents the cutoff value. The data are presented as the means ± SEMs (six–seven rats per group). Intergroup differences were analyzed using ANOVA with Bonferroni’s multiple comparisons post hoc test. # *p* < 0.05, ## *p* < 0.01, ### *p* < 0.001 indicate a significant difference between V- and PHT-treated CCI-exposed animals; @@ *p* < 0.01, @@@ *p* < 0.001 indicate differences before (pretest) and 1 h after V/PHT administration; ^ *p* < 0.05 indicates differences between PHT 10 mg/kg compared to PHT 60 mg/kg; ^^^ *p* < 0.001 indicates differences between PHT 10 mg/kg compared to PHT 30 mg/kg; Abbreviations: V, vehicle; PHT, phenytoin; CCI, chronic constriction injury.

Single *i.p.* injections of PHT at doses of 10, 30 and 60 mg/kg were administered on Day 7 after CCI in rats. The influence on hypersensitivity to tactile and thermal stimuli was measured using von Frey (Figure 1A) and cold plate (Figure 1B) tests, respectively, at 1 h after drug administration. PHT at doses of 30 mg/kg and 60 mg/kg showed strong antinociceptive effects, diminishing the CCI-evoked hypersensitivity to tactile and thermal stimuli as measured by von Frey (Figure 1A) and cold plate (Figure 1B) tests, respectively. We also demonstrated that this drug at a dose of 10 mg/kg had strong significant antinociceptive effects (*p* < 0.001), attenuating tactile (Figure 1A) and thermal hypersensitivity (Figure 1B) 1 h after injection in both tests. In the von Frey test measuring tactile hypersensitivity, we observed a significantly better antinociceptive effect of PHT at doses of 30 mg/kg (*p* < 0.001) and 60 mg/kg (*p* < 0.05) than at a dose of 10 mg/kg (Figure 1A). Because it had the strongest antinociceptive effect, the dose of 30 mg/kg was selected for chronic *i.p.* PHT administration as well as for the combined regimen of PHT and M.

### 3.2. The Influence of Repeated i.p. Phenytoin Administration on Pain-Related Behavior Measured on Days 2 and 7 after Chronic Constriction Injury of the Sciatic Nerve in Rats

**Figure 2:**
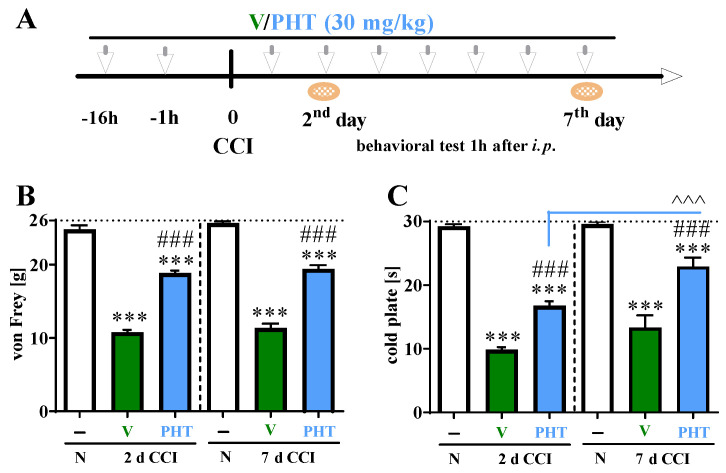
Effect of repeated administration of PHT (30 mg/kg; intraperitoneally (**A**)) 16 h and 1 h before CCI and then once a day for 7 days on pain-related behaviors in rats [von Frey test—(**B**) and cold plate test—(**C**)] 1 h after drug administration on Days 2 and 7 after chronic constriction injury of the sciatic nerve. The horizontal dotted line represents the cutoff value. The data are presented as the means ± SEMs (12 rats per group). Intergroup differences were analyzed using ANOVA with Bonferroni’s multiple comparisons post hoc test. *** *p* < 0.001 indicates significant differences between the control group (naive animals) and V- or PHT-treated CCI-exposed rats; ### *p* < 0.001 indicates significant differences between V- and PHT-treated CCI-exposed rats. ^^^ *p* < 0.001 indicates differences between PHT 30 mg/kg on Day 2 after CCI and PHT 30 mg/kg on Day 7 after CCI. Abbreviations: N, naive; V, vehicle; PHT, phenytoin; CCI, chronic constriction injury.

All CCI-exposed rats developed tactile (*p* < 0.001; Figure 2B) and thermal (*p* < 0.001; Figure 2C) hypersensitivity relative to the I group on Day 2 and 7 following CCI. Preemptive and repeated (for 7 days) *i.p.* PHT (30 mg/kg) administration (Figure 2A), compared with vehicle treatment at both Day 2 and Day 7, significantly reduced tactile and thermal hypersensitivity in the von Frey (Figure 2B) and cold plate (Figure 2C) tests, respectively. A significantly increased antinociceptive effect of repeated *i.p.* administration of PHT was observed on Day 7 post-CCI compared to Day 2 post-CCI in the cold plate (*p* < 0.001) test (Figure 2C).

### 3.3. The Influence of Repeated i.p. Phenytoin Administration on Microglia/Macrophage Marker Measured on Day 7 after Chronic Constriction Injury of the Sciatic Nerve in Rats

**Figure 3:**
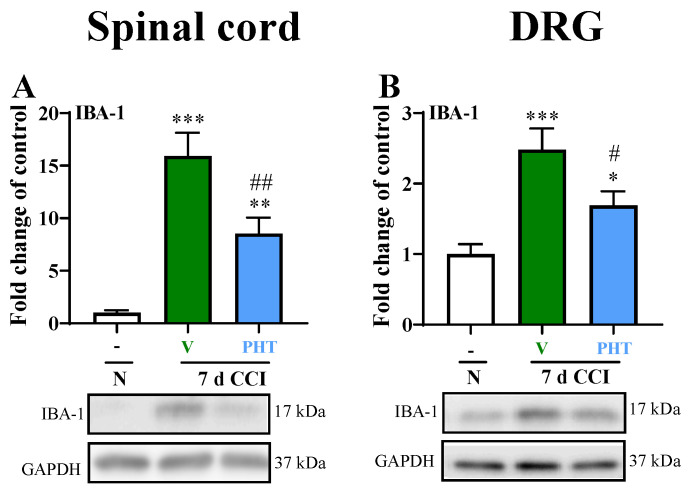
The effect of repeated intraperitoneal administration of PHT (30 mg/kg; intraperitoneally, 16 h and 1 h before CCI and then once a day for 7 days) on the IBA-1 in the spinal cord (**A**) and DRGs (**B**) on Day 7 after chronic constriction injury of the sciatic nerve. The data are presented as the means ± SEMs (7–15 rats per group). Intergroup differences were analyzed using ANOVA with Bonferroni’s multiple comparisons post hoc test. * *p* < 0.05, ** *p* < 0.01, *** *p* < 0.001 indicate significant differences between the control (naive) and V- or PHT-treated CCI-exposed rats; # *p* < 0.05, ## *p* < 0.01, indicate significant differences between V- and PHT-treated CCI-exposed rats. Abbreviations: N, naive; V, vehicle; PHT, phenytoin; CCI, chronic constriction injury.

In the spinal cord, a strong increase in IBA-1 levels was observed on Day 7 in CCI-exposed rats compared with naive rats. Preemptive and repeated (for 7 days) *i.p.* PHT (30 mg/kg) administration significantly decreased the changes in microglia/macrophage marker (*p* < 0.01; Figure 3A) in the spinal cord. Moreover, similarly in the DRGs, an increase in IBA-1 protein levels after CCI was detected, and PHT effectively diminished these changes (*p* < 0.05, Figure 3B).

### 3.4. The Influence of a Single i.p. Administration of Phenytoin on the Efficacy of Morphine on Day 8 after Chronic Constriction Injury of the Sciatic Nerve in Rats

**Figure 4:**
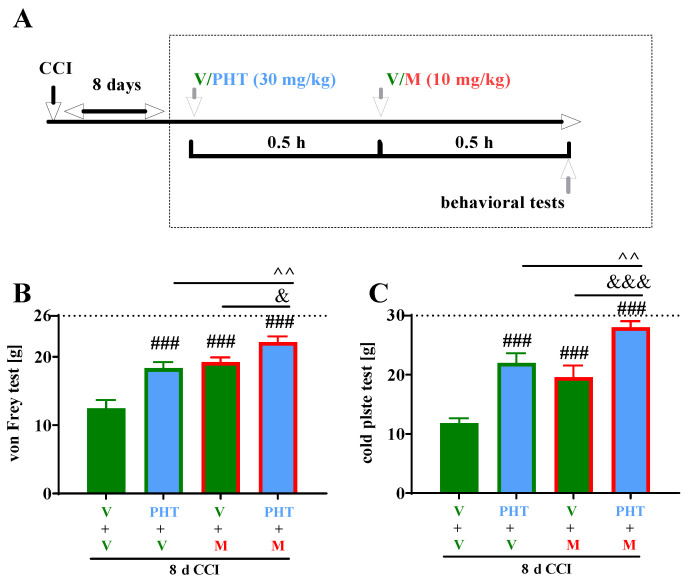
Effect of a single administration of PHT (30 mg/kg; intraperitoneally (**A**)) on pain-related behaviors in rats (von Frey test—(**B**) and cold plate test—(**C**)) and the analgesic effects of M (10 mg/kg; intraperitoneally (**B**,**C**)) 1 h after vehicle or PHT administration (0.5 h after opioid injections) on Day 8 after chronic constriction injury of the sciatic nerve The data are presented as the means ± S.E.M.s (7–8 rats per group). The results were analyzed using one-way ANOVA with Bonferroni’s post hoc multiple comparisons test. ### *p* < 0.001 indicates differences compared with V-treated CCI-exposed rats (V+V); & *p* < 0.05, &&& *p* < 0.001 indicate differences between the V+M and PHT+M-treated CCI-exposed rats; ^^ *p* < 0.01 indicates differences between the PHT+V- and PHT+M-treated CCI-exposed rats. Abbreviations: V, vehicle; M, morphine; PHT, phenytoin; CCI, chronic constriction injury.

The effects of a single administration of PHT (at a dose of 30 mg/kg) on morphine effectiveness were studied on Day 8 in CCI-exposed rats (Figure 4A) using von Frey and cold plate tests (Figure 4B,C). A single *i.p.* PHT injection produced a significant antinociceptive effect compared with vehicle treatment in the von Frey (*p* < 0.001) (Figure 4B) and in cold plate tests (*p* < 0.001) (Figure 4C). A single *i.p.* injection of M (10 mg/kg) on Day 8 following CCI-attenuated neuropathic pain behavior in rats in both tests, and the antinociceptive effect of M was comparable to that of PHT at a dose of 30 mg/kg (Figure 4B,C). Moreover, PHT diminished thermal hypersensitivity to a similar extent to M (Figure 4C). Nevertheless, the combined administration of PHT and M resulted in a significantly more enhanced antinociceptive effect following tactile (*p* < 0.01 compared to PHT and *p* < 0.05 compared to M) and thermal (*p* < 0.01 compared to PHT and *p* < 0.001 compared to M) stimulation than the administration of either drug alone (Figure 4B,C).

## 4. Discussion

In the present study, we demonstrated that single (10, 30 and 60 mg/kg) and repeated (30 mg/kg) *i.p.* administration of PHT to Wistar rats strongly reduced tactile and thermal hypersensitivity in animals subjected to chronic constriction injury of the sciatic nerve. Consistent with our results, Guevara-Lopez et al. [63] observed a reduction in hypersensitivity in Wistar rats in a partial sciatic nerve ligation (PSNL) model after repeated *i.p.* administration of PHT at a dose even lower than that used in our study (7.5 mg/kg). However, in Sprague Dawley rats, it has been shown that a single administration of PHT at a dose of 10 mg/kg *i.p.* did not reduce hypersensitivity in the PSNL model [56]. Similar results were obtained in a CCI model by Ko et al. [64] in Sprague Dawley rats, in which only a very high dose of PHT (100 mg/kg) attenuated neuropathic pain symptoms. The above-mentioned results seem to be inconsistent; however, in our opinion, they are rat strain dependent—more studies are certainly needed. Additionally, the good analgesic potential of intravenous (*i.v.*) injections of the prodrug of PHT, fosphenytoin (fPHT, 15, 30 mg/kg) was shown in a mouse model of acute herpes zoster infection affecting peripheral nerves [65]. In that study fPHT administered *i.v.* reduced hypersensitivity and furthermore diminished the spontaneous duration of paw licking, which led researchers to conclude that fPHT may be a viable option in the clinic for patients with acute herpes zoster infection, especially for spontaneous pain treatment [65].

Moreover, other studies have shown that in inflammatory pain models, the effective doses of PHT are much lower than those in neuropathic pain models, which is also typical for other analgesic drugs, including morphine [66,67]. For example, it was shown in a formalin mouse model that PHT administered at a dose of 6 mg/kg *i.p.* reduced phase II of pain behavior [56]. Similarly, in a visceral mouse pain model, PHT at the same dose diminished the abdominal constriction response [56]. Consistent with these findings, de Queiroz et al. [68] showed that hydantoin, which is chemically and structurally similar to PHT, reduced pain behavior in phase II of the formalin test and the number of writhes in a mouse model of visceral pain. In acute pain models, *i.p.* PHT at doses of 2.5–25 mg/kg significantly attenuated thermal hypersensitivity in a dose-dependent manner [69]. The data from our study together with the cited literature confirm the effectiveness of systemic PHT in neuropathic pain symptoms, making this drug a viable option for use in clinical practice; however, more studies are necessary to assess the effective analgesic dose of PHT.

In the present study, we demonstrated for the first time that repeated *i.p.* administration of PHT at a dose of 30 mg/kg did not induce any tolerance to the analgesic effects of PHT to tactile and thermal stimuli in a CCI rat model during 7 days of observation. To date, there are no data available referring to the development of tolerance after repeated administrations of PHT in pain models. The only data come from a rat model of epilepsy, and they demonstrated a lack of tolerance development to the anticonvulsant effect of PHT (75 mg/kg) recorded during 2 weeks of treatment; however, tolerance appeared to adverse effects such as motor impairment and hypothermia [70]. Our study provided the first evidence of even a slight increase in the antinociceptive effect of PHT on thermal, but not tactile, stimuli on Day 7 compared to Day 2 after CCI. The first observations that thermal nociception is more sensitive than mechanical nociception to agents blocking Nav came from the study of Sakaue et al. [69] in an acute pain model in mice. In this pain model, Nav blockers such as PHT, carbamazepine, mexiletine and lidocaine reduced the sensitivity to heat noxious stimuli better than the sensitivity to mechanical stimuli, in contrast to morphine, which produced similar antinociceptive effects on thermal and mechanical stimuli. The authors attributed this effect to the different sensitivity of nociceptive neurons to substances with local anesthetic activity, indicating that thin, non-myelinated subtypes of C fibers activated by thermal stimuli are more sensitive to Nav blockers than Aδ and C fibers transmitting mechanical nociceptive signals [71]. However, it remains unclear whether the improvement in the antinociceptive effect of PHT on reducing thermal sensitivity in our study was derived from the Nav blockade produced by the drug; however, the crucial role of Nav1.6 in thermal hypersensitivity has been confirmed by Deuis et al. [72]. Moreover, PHT is suggested to block Nav poorly at slow firing rates but suppresses the high-frequency repetitive firings [20], i.e., coming from the pathologically changed function of Nav following neuronal injury (e.g., abnormal firing patterns, and ectopic activity) [73]. We cannot exclude the anti-inflammatory mechanism of action of PHT in improving the reduction in thermal sensitivity observed in our study on Day 7 after CCI. Silencing the microglia by repetitive administration of PHT and indirect influence or modulation of several neuroimmunological factors by PHT has been confirmed in a rat epilepsy model [49]. The involvement of selected neurotrophic factors produced by immunocompetent cells together with corresponding receptors (glial cell line-derived neurotrophic factor/GFRα3, nerve growth factor/TrkA) in cold sensitization through coexpression with transient receptor potential melastatin (TRPM8) and the upregulation of transient receptor potential ankyrin (TRPA1) has been demonstrated in in vivo animal studies [74,75]. The influence of PHT on VGCCs should also be considered, since the role of N-type VGCCs in thermal hyperalgesia has been demonstrated in an in vitro study by Caminski et al. [76]. In sum, we can only hypothesize how PHT could enhance the antinociceptive effect observed on Day 7 in our study. In models of neuropathic pain, several mechanisms are involved, creating an extremely complex interplay between different types of cells and a myriad of neurotransmitters, both excitatory and inhibitory, which could be influenced by PHT. Whether systemic PHT would be more effective in patients with neuropathic pain with abnormal sensitivity to thermal compared to mechanical stimuli remains unclear and needs further assessment in clinical studies.

In our study, we demonstrated that a single *i.p.* injection of PHT at a dose of 30 mg/kg or M at a dose of 10 mg/kg produced comparable antinociceptive effects to either tactile or thermal stimuli on Day 8 after CCI, when hypersensitivity was fully developed in the studied rat model. The applied *i.p.* dose of 10 mg/kg M in a NP model showed relatively weak analgesic effects, which is consistent with previously published results [60], whereas the same dose in inflammatory pain provides very strong pain relief [77]. The literature data clearly indicate that, similar to what is observed in the clinic [3], M partially loses its effectiveness in NP states when compared to nociceptive and inflammatory acute pain. A similar phenomenon was observed for PHT [56,69] but to a lesser extent than for morphine. The reduced effectiveness of analgesic drugs may be partially explained by the full development of peripheral and central sensitization on Day 8 after CCI, together with their underlying cellular mechanisms, resulting in strong tactile and thermal hypersensitivity [78,79]. The literature data indicate that the PHT mechanism of action is multitargeted and involves different molecular mechanisms than M, suggesting that their combined administration may have beneficial effects. The antinociceptive effect of PHT on tactile and mechanical sensitivity in NP may be attributed to its influence not only on Nav but also on VGCC and anti-inflammatory factors [34]. The involvement of multiple antinociceptive mechanisms of PHT may explain its prolonged analgesic effects after infusion, as demonstrated by McCleane in humans [15], i.e., a significant reduction in shooting pain until Day 5 after infusion, in sensitivity to Day 3 and in overall pain scores on Day 1 when compared with pre-infusion scores in patients with refractory NP.

Although the antinociceptive properties of PHT have already been confirmed in some experimental studies of pain of varying etiologies, the neuroimmunological background of its action remains unclear; hence, we chose it as the subject of our research. According to recent evidence, the activation of microglia/macrophages is critical for the development and maintenance of neuropathic pain [2,50,51]. We have already shown by Western blot analysis that the activation of Iba-1-positive cells in the spinal cord begins soon after injury and is increased on Day 2, but the greatest changes are observed from Day 7 to Day 14 [80]. In addition, by immunohistochemical analysis, we have shown that microglial morphology changes after sciatic nerve injury, from the typical resting phenotype seen in the spinal cord of naïve rats into the enlarged and amoeboid form [81]. Since previously published studies demonstrated the strongest spinal upregulation of microglia and macrophages markers around Days 7–8 after CCI [81,82], we decided to examine the effect of repeated *i.p.* PHT administrations on Day 8 after CCI. Importantly, in our study, we observed enhanced levels of activation, proliferation and/or infiltration of the microglia/macrophage marker IBA-1 on Day 7 after CCI and noticed that repeated *i.p.* administration of PHT at a dose of 30 mg/kg prevented the activation, proliferation and/or influx of microglia/macrophages into the spinal cord and DRG. Our findings support the hypothesis that PHT administration affects the activity of microglia and macrophages, key players in the pathogenesis of neuropathic pain [51,82,83,84]. In the early 2000s, it was confirmed that the blockade of Nav 1.6 by PHT diminished the activation, proliferation and/or phagocytosis of CD45+ cells, including microglia, and protected axons in an animal model of multiple sclerosis and experimental acute encephalomyelitis (EAE) [41,42,44]. Recently, experimental studies have assessed the impact of PHT on glia in models of epilepsy [49] and in the in vitro studies [24,47]. First, it was found that PHT could diminish by 50% the release of pro-inflammatory cytokines (Il-1α, IL-1β, TNFα) from activated rat microglia in the in vitro study [24]. Later, in the in vitro study of Dambach et al. [47], using an astroglia/microglia coculture model of inflammation, it was proven that PHT increased the level of pro-inflammatory TNFα but simultaneously also the anti-inflammatory TGF-β1, much more so than gabapentin. The study of Pottoo et al. [49] in a rat model of epilepsy demonstrated that repeated oral administration of PHT (20, 40 mg/kg) significantly reduced the levels of proinflammatory cytokines such as IL-1β, IL-6 and TNFα in the hippocampus. However, whether the repeated *i.p.* administrations of PHT in a neuropathy model would have similar effects certainly needs further assessment [49]. Cytokines are crucial in inflammation during epilepsy development but also play an important role in pain development [51,84,85] and attenuate morphine analgesia [85,86,87,88,89,90,91]. Importantly, it was also published that in a rat model of wound healing, PHT helped to reduce tissue edema and decrease inflammatory cell infiltration [46]. Moreover, in vitro, in human overgrown gingival tissue, lower levels of inflammatory infiltrates were shown after exposure to PHT [43]. Importantly, the literature data and ours unambiguously indicate that the inhibition of microglia/macrophage activation at the level of the spinal cord and DRGs consequently contributes to the reduction in the secretion of proinflammatory factors, which often have pronociceptive and anti-opioid effects, such as the above-mentioned cytokines IL-1β, IL-6 and TNFα [51,80,82,84,85,86,87]. Based on our research and others, further behavioral and biochemical experiments using different routes of repeated administration of PHT alone and in combination with opioids are certainly needed.

Importantly, our studies provided the first evidence that a single *i.p.* administration of PHT at a dose of 30 mg/kg on Day 8 after CCI significantly enhanced morphine-induced antinociception in rats. We conducted a review of the literature but found no papers dealing specifically with the antinociceptive effect of PHT co-administered with opioids in animal pain models. In theory, the combination of PHT and opioids could work due to their different mechanisms of action and different influences on thermal and mechanical pain thresholds, which has been demonstrated by Sakaue et al. [69] in an acute pain model in mice. However, thus far, we have found only two papers demonstrating the beneficial analgesic effect of opioids combined with PHT in patients with cancer-related pain. In a randomized, double-blind study by Yajnik et al. [8], coadministration of buprenorphine 0.1 mg twice daily sublingually with oral PHT 50 mg twice daily provided better pain relief than buprenorphine 0.2 mg twice daily alone or PHT 100 mg twice daily alone, with a better safety profile than buprenorphine alone. However, the small number of patients included, the lack of statistically significant differences between the groups studied, and the lack of reporting on the neuropathic component of pain are the main limitations of that study. Importantly, Chang [9] presented a case report of a patient with severe cancer-related pain diagnosed with lumbosacral plexopathy that was effectively treated with oral PHT 100 mg twice daily together with oral morphine 540 mg/day. Further evidence for the effectiveness of the combination of opioids and antiepileptics targeting Nav in patients with neuropathic pain is limited. Experts analyzing the available literature emphasize that in real-world clinical practice, anticonvulsants acting on Nav are often combined with opioids to manage trigeminal neuralgia [92], despite limited clinical evidence. Experiments in animal pain models have demonstrated that Nav blockers such as carbamazepine potentiate the morphine analgesia of postoperative pain in morphine-dependent rats [93] and in model of neuropathic pain [94]; that intrathecally co-administered lamotrigine attenuates antinociceptive tolerance to morphine in rats [95]; and that topiramate and lamotrigine synergistically interact with tramadol in a nociceptive pain model [96]. The potential molecular mechanisms by which anticonvulsants enhance opioid-induced analgesia may involve the blockade of overexpressed and overactive Nav1.7 in injured neurons [97] and Nav 1.6 in microglia [24]. Moreover, the activation of neuronal and glial toll-like receptor 4 (TLR4) by morphine and its metabolite morphine-3-glucuronide may additionally contribute to the pathological function of several subtypes of Nav [97] and contribute to the decreased effectiveness of morphine in neuropathic pain models. Thus, PHT, by silencing the activated microglia and reducing abnormal activity of Nav in injured neurons, may enhance the antinociceptive effect of morphine observed in our study. Our previous experiments indicate that silencing microglia through pentoxifylline and minocycline decrease microglial activation, thereby suppressing the development of neuropathic pain and improving morphine analgesia [51,87,98]. Irrespective of the mechanisms involved, our study confirmed that PHT may be co-administered with morphine to achieve better antinociception in neuropathic pain, which can be clearly translated into clinical practice.

## 5. Conclusions

In summary, we found evidence in our study for the efficacy of systemically administered PHT in the treatment of neuropathic pain. The drug decreased tactile and thermal hypersensitivity after repeated or even single administrations once neuropathic pain symptoms fully developed. Moreover, the antinociceptive effect on thermal stimuli increased after repeated administrations of PHT, which could possibly make the drug more suitable for patients with abnormal sensitivity to thermal rather than mechanical stimuli. Our findings suggest that several indirect mechanisms may be responsible for the analgesic action of PHT, not just its interactions with sodium and calcium channels, as was already suggested by others [72,76], which eventually confirms the pleiotropic character of PHT. We are the first to report that PHT diminished microglia/macrophage activation and/or infiltration at the spinal cord and DRG levels 7 days after nerve injury, and it reduced pain behaviors. Importantly, PHT administered with M evoked better antinociception than either drug injected alone. In our opinion, in clinical practice, patients with acute and refractory neuropathic pain, particularly with hypersensitivity to thermal stimuli, may benefit from PHT administration. Our findings support the hypothesis that pharmacological improvement of opioid analgesia through PHT coadministration may represent a more effective multimodal strategy in neuropathic pain management.

## Data Availability

The data presented in this study are available on request from the corresponding author.
